# Induction of DNA Demethylation: Strategies and Consequences

**DOI:** 10.3390/epigenomes9020011

**Published:** 2025-04-12

**Authors:** Pietro Salvatore Carollo, Viviana Barra

**Affiliations:** Department of Biological Chemical and Pharmaceutical Sciences and Technologies, University of Palermo, 90128 Palermo, Italy

**Keywords:** DNA methylation, DNA demethylating drugs, DNMT1, AID, TET enzymes, CRISPR-Cas9

## Abstract

DNA methylation is an important epigenetic modification with a plethora of effects on cells, ranging from the regulation of gene transcription to shaping chromatin structure. Notably, DNA methylation occurs thanks to the activity of DNA methyltransferases (DNMTs), which covalently add a methyl group to the cytosine in position 5′ in CpG dinucleotides. Different strategies have been developed to study the effects of DNA methylation in cells, involving either DNMTs inhibition (passive DNA demethylation) or the use of Ten-eleven translocation protein (TET) family enzymes, which directly demethylate DNA (active DNA demethylation). In this manuscript, we will briefly cover the most commonly used strategies in the last two decades to achieve DNA demethylation, along with their effects on cells. We will also discuss some of the newest inducible ways to inhibit DNMTs without remarkable side effects, as well as the effect of non-coding RNAs on DNA methylation. Lastly, we will briefly examine the use of DNA methylation inhibition in biomedical research.

## 1. Introduction

**Figure 1:**
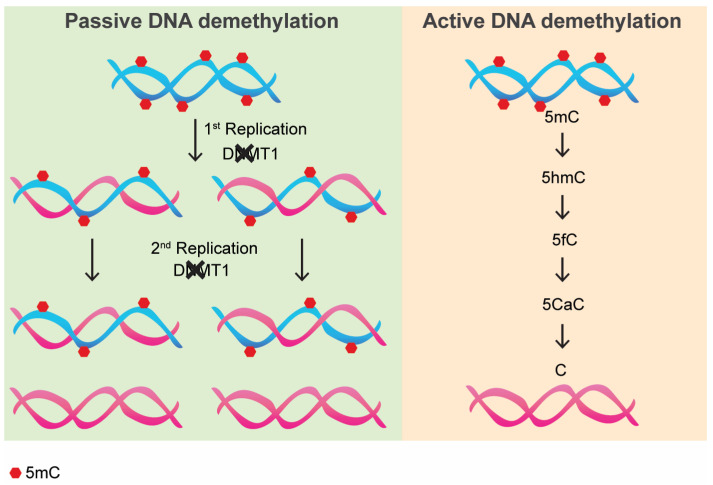
Passive vs. Active DNA demethylation. Red hexagons represent methyl groups on 5′ carbon of cytosine ring. Blue DNA strands represent methylated strands, whereas red strands represent strands that have lost methylation. 5mC: 5′methyl-cytosine; 5hmC: 5 hydroxymethyl-cytosine; 5fC: 5-formylcytosine; 5CaC: 5-carboxylcytosine; C: cytosine.

Chromatin condensation in cells, apart from histone modifications, relies also on DNA methylation on CpG dinucleotides, a process of pivotal importance involved in gene regulation, development, senescence, and chromatin/nucleus dynamics and mechanics [1,2,3,4,5,6,7,8,9,10,11]. DNA methyltransferases (DNMTs) add a methyl group to 5′ position in cytosine within CpG sites, thus generating 5′methyl-cytosine (5mC). Methylated DNA has an increased stiffness, resulting in a more condensed chromatin that favors its heterochromatic state. DNMTs can canonically methylate either de novo, as DNMT3A and DNMT3B do by methylating un-methylated DNA, or to maintain 5mCs by copying the pattern on the newly synthesized DNA strand at each S-phase of the cell cycle, as DNMT1 does [12]. It has been demonstrated that, upon pharmacological as well as genetic inhibition of DNMT1, “passive demethylation” occurs. This is a phenomenon whereby DNA methylation at CpG dinucleotides is gradually diluted upon subsequent cell divisions due to the absence of the maintenance methylation [13] (Figure 1, left). Recently, whether an “active” process of DNA demethylation (Figure 1, right) could exist has been questioned. To date, there is no evidence for a “direct” process of DNA demethylation. However, it has been demonstrated that the removal of a CpG methyl group can take place as a multi-step process, whereby Ten-eleven translocation protein (TET) family enzymes (TET1, TET2, and TET3) together with thymine DNA glycosylase (TDG) are involved [13]. Studying the effect of DNA demethylation is paramount, since most human cancers are associated with re-expression of oncogenes, whose regulatory sequences are normally hypermethylated in non-cancerous cells [14]. Thus, it is clear that a strategy to study the effects of DNA demethylation “on demand” is essential, and this has also been made possible thanks to the latest advancement in gene editing technology.

In this manuscript, we will review the literature in which different approaches to induce DNA demethylation have been used, ranging from genome-wide demethylation via genetic perturbation and pharmacological inhibition of DNMT1, to gene-locus specific demethylation via modulation of TET enzymes and CRISPR-Cas9 technology. We will also discuss the role of non-coding RNAs in regulating DNMTs’ activity.

## 2. Passive DNA Methylation: How It Works and Its Consequences

Generally speaking, passive DNA demethylation can be acquired by either pharmacological inhibition of DNMT1 activity through demethylating drugs, or *DNMT1* gene knockout/knockdown, as well as DNMT1 protein inducible degradation. Pharmacological inhibitors, such as 5-Azacitidine (AZA) and 5-aza-2′-deoxycytidine (DAC, also known as Decitabine), have been widely used to induce global CpG demethylation in cells [15,16]. However, more recently, the selective DNMT1 inhibitor GSK3685032 has been discovered. By intercalating into DNMT1-bound hemi-methylated DNA, GSK3685032 induces DNA demethylation, as a consequence of genome-trapped DNMT1 degradation [17,18]. On the other hand, AZA and DAC are cytidine analogs that can be incorporated into the DNA during replication. From this, the formation follows of drug/DNMT1 adducts in the DNA, thus sequestering DNMT1 and lowering its protein levels [19]. The latter can thus be the consequence of ubiquitin-E3 ligase induction, which allows proteasome degradation of free DNMT1 molecules, preferentially [20]. While AZA is a ribonucleoside and is incorporated in both RNA and DNA, potentially impairing protein translation [21,22], DAC is a deoxyribonucleoside and is incorporated in DNA only. Both drugs induce DNMT1 impairment with consequent DNA hypomethylation and DNA damage, but with some differences. Indeed, Hollenbach et al. showed that, in a panel of acute myeloid leukemia (AML) human cells, AZA was more potent in inducing cell viability reduction at high concentrations compared to DAC. This occurred because AZA is predominantly incorporated into RNA throughout the cell cycle, whereas DAC is incorporated into DNA in S-phase only. Higher cellular lethality with AZA was related to its capability to generally impair protein synthesis, differently from DAC. However, both drugs did deplete DNMT1, which led to global DNA demethylation and DNA damage induction, with DAC triggering these effects at lower concentrations compared to AZA. In addition, both AZA and DAC impaired cell cycle progression but in different ways: AZA promoted subG1 accumulation, whereas DAC, apart from enriching the subG1 phase, caused cells to accumulate in the G2/M phase. Both drugs also increased apoptosis. Finally, AZA and DAC treatments were found to induce transcription of different genes, being part of specific cellular pathways: genes related to both metabolism and cell cycle for AZA treatment and genes related to cell cycle differentiation for DAC treatment. Thus, multiple pathways were involved in reducing cell viability, making it difficult to understand the primary effect of DNA methylation loss itself [15].

Apart from inducing hypomethylation and DNA damage, demethylating drugs can also trigger aneuploidy. Indeed, Costa and colleagues found that treating near diploid HCT116 (colorectal cancer) cells with DAC caused hypomethylation of chromosomes’ pericentromeric regions, which resulted in aneuploidy and mitotic defects. Thus, DNMT1 activity is important to avoid aneuploidy. Indeed, HCT116 cells treated for up to 72 h with DAC displayed a time-dependent 5mC reduction. This was associated with increasing aneuploidy, which was a consequence of the methylation loss. In addition, sister chromatid cohesion was impaired, and railroad track chromosomes were observed. DAC also caused mitotic defects and a slowdown of anaphase onset [16].

Apart from chemical inhibition, DNMT1 has been disabled by both gene knockout via Cre-recombinase or simply by homologous recombination, either in cells [23,24] or in animals [25], and by RNA interference (RNAi) [26]. For instance, Rhee et al. used Cre-recombinase to disrupt exons 3, 4, and 5 in the *DNMT1* gene sequence in HCT116 cells, thus obtaining DNMT1+/− and DNMT1−/− genotypes. This led to impairment of from 66% (DNMT1+/−) to 96% (DNMT1−/−) of methyltransferase activity in DNMT1, even though the global amount of 5mC had a milder decrease (20%). Interestingly, methylation loss occurred at specific juxta-centromeric sequences rather than all over the genome [23].

Later, Gaudet and colleagues generated mice harboring one allele of the *DNMT1* gene hypo-morphic (DNMT1chip) and the other one null. This condition was compatible with life, contrary to DNMT1 double knockout. DNMT1chip/- mice displayed a dramatic reduction of genome-wide DNA methylation, which resulted also in tumor formation [25].

DNMT1 knockout can also lead to cell cycle arrest and cell death. In this regard, Chen et al. induced DNMT1 depletion via Cre-recombinase in HCT116 cells. Specifically, by Cre-recombinase induction, exons 30–33, spanning the DNMT1 catalytic domain, were removed on one allele, while the other one had been previously knocked out by homologous recombination. As a consequence, DNMT1 protein was not detected, and this was accompanied by a global reduction of DNMT1 methyltransferase activity. In addition, cells with no DNMT1 displayed aberrant morphology, were binucleated, had aberrant chromosomes, underwent G2 arrest, presented DNA damage with activation of p53/p21 axis, and underwent cell death. However, 5mC was only reduced by 10% [24].

DNMT1 transient depletion has also been shown to trigger cell cycle arrest and aneuploidy in human cells. Indeed, Barra et al. demonstrated that depletion of DNMT1 for 72 h caused a slowdown in cell proliferation, which was higher in IMR90 fibroblasts compared to HCT116 cells. This reduction in proliferation was caused by a G1-phase block for IMR90 cells and an S-phase accumulation for HCT116 cells. In the case of IMR90 cells, G1 arrest was triggered by p14/p53/p21 axis activation, which was not related to DNA damage. Depletion of DNMT1 caused a global reduction of 5mC in HCT116 cells. This was also achieved in IMR90 cells only when G1 arrest was bypassed upon double silencing of either DNMT1/p53 or DNMT1/p14 [26].

Recently, CRISPR-Cas9 technology has been used to inhibit *DNMT1* gene transcription, which thus results in global DNA demethylation in RPE-1 cells [27]. Indeed, Besselink et al. fused the gene sequence of *KRAB*, a transcriptional repressor, to the gene sequence of a deactivated Cas9 (*dCas9*) in RPE-1 cells. This chimeric construct (dCas9-KRAB) was then able to be recruited to the transcription starting site of the desired target gene (*DNMT1*) by a specific single-guide RNA (sgRNA), whose transcription is induced by the addition of doxycycline. Doxycycline addition decreased DNMT1 transcript as well as its protein levels. This reduction was followed by a genome-wide decrease of 5mC at the level of genes, promoters, and CpG islands (CGI). Genome-wide demethylation also allows cell nuclei to acquire an aberrant morphology (kidney-shaped morphology). In addition, loss of DNA methylation triggered aneuploidy and the methylation level of pericentromeric DNA was decreased. Global loss of DNA methylation is a marker of genome instability and, as a consequence, of tumorigenesis. Thus, the authors concluded that the system they generated is feasible for studying DNA methylation loss and its impact on genome stability [27].

Apart from pharmacological inhibition and genetic perturbations, a new strategy has been recently designed to degrade DNMT1 protein [28]. Specifically, in DLD-1 or RPE1- cells stably expressing a MYC-tagged osTIR1 (a plant E3 ubiquitin ligase), the endogenous DNMT1 alleles were edited to have the sequences of the Neon green fluorescent protein and of the Auxin-inducible degron (AID) added in frame at the 5′ terminus. In the presence of the plant hormone auxin, DNMT1 protein was rapidly degraded, and this degradation was totally reversible upon auxin washout. It is worth noting that such a DNMT1 construct did not alter genome-wide methylation status, which was not distinguishable from cells carrying wild-type DNMT1. Upon induction of DNMT1 degradation, there was a gradual genome-wide reduction of 5mC, and this reduction was reversed upon 4 days of auxin washout. Reduction of methylation at Alu and Satellite II repetitive sequences was also noticed. Depletion of DNMT1 led DLD-1 and RPE-1 cells to arrest in G1 phase, which was mirrored by a decreased clonogenic capacity in both cell lines. However, upon washout, clonal capacity of DLD-1 cells was recovered. Upon DNMT1 degradation in DLD-1 cells, the deposition of the heterochromatin markers H3K9me3 and H3K27me3 was also influenced, with rearrangement from the nuclear periphery to the nucleoplasm. This latter was reversed upon 4 days of washout [28]. Inducible DNMT1 degradation via AID/Tir1 system allows an immediate loss of DNMT1 protein, which thus results in passive DNA demethylation. This occurs without major side effects that can be related to the use of drugs with pleiotropic effect or to the adaption to a new genetic condition, e.g., upon knockdown/knockout strategies.

Very recently, our group, by using the same AID system as reported in [28], has shown that, in the non-cancerous cell line RPE1, auxin-induced degradation of DNMT1 causes cell cycle block between G1/S phases. This block is due to the activation of the p53/p21 axis, and it is related to the global reduction of 5mC, which reaches its peak at 4 days of auxin treatment. However, upon auxin washout, cells can re-enter the cell cycle. This means that DNMT1 recovery is sufficient to rescue the cell cycle block, thus suggesting that cells can safely proliferate if DNA methylation levels, even minimal, are maintained [29].

## 3. Active DNA Demethylation: How It Works and Its Consequences

Active DNA demethylation is promoted by the orchestrated activity of TET enzymes, which first oxidize 5mC to 5-hydroxymethyl cytosine (5hmC), then to 5-formylcytosine (5fC) and, lastly, to 5-carboxylcytosine (5caC). Then, TDG can remove 5fC or 5caC, thus generating an abasic site, a common DNA lesion promptly repaired. To this, the activation of the Base Excision Repair (BER) follows, which corrects the lesion, restoring the 5-cytosine without introducing DNA double-strand breaks (DBDs) [13,30]. Another mechanism, though less characterized, has been proposed for active DNA demethylation. This involves the deamination of 5mC or 5hmC to thymidine or 5-hydroxymethyl uracil, respectively. T:G and 5hmU:G mismatches are, thus, generated and are recognized by TDG or methyl-binding domain 4 (MBD4) [31,32], leading to BER [33].

Some evidence suggests other active DNA demethylation mechanisms, not involving either TET or TDG/BER, through which direct conversion of 5mC to 5caC is possible. The enzymes which have this capability belong to the AlkB homolog (ALKBH) superfamily: the 5caC they generate may be used as a subsequent substrate by TDG to complete the conversion to cytosine (C) [13].

In addition, following 5hmC, there can be the generation of a C via the passive dilution mechanism, which takes into account the “dispersion” of the modification on C after several rounds of DNA replication [13,30].

There are multiple strategies to directly bring TET enzymes to the gene sequences to be demethylated, and these strategies rely on the use of recombinant transcription activator-like effector (TALE) repeats [34], as well as zinc finger (ZF) domains [35] or CRISPR-Cas9 technologies [36].

TALE repeats were used in a construct by Maeder and colleagues to demethylate CpG dinucleotides in specific promoter sequences. This construct took advantage of the TET1 catalytic domain (TET1CD) as well as customized TALE repeats, DNA binding domains able to bind a desired DNA sequence. When TALE repeat arrays KL1 and KL2 were fused with TET1CD, CpG dinucleotides in the promoter region of *KLF4* human gene were demethylated upon transfection in human erythroleukemic K562 cells. In addition, the demethylation of the promoter CpG dinucleotides was also associated with increased transcription of *RHOFX2* gene, normally silent in non-germline cells, in both HeLa and HEK-293 cells expressing TALE repeat arrays RH3 and RH4 fused with TET1CD [34].

Demethylation of promoter CpG dinucleotides coupled with increased gene expression was also achieved with TALE–TET1CD constructs targeting human beta-globin (*HBB*) gene promoter, upon transfection in K562 cells. Demethylation efficiency was dependent on each single TALE repeat array used. Maeder et al. also fused ZF sequences with TET1CD and obtained similar results to TALE–TET1CD constructs [34].

Apart from TALE repeats, as stated before, ZF domains can be also used to directly bring the catalytic domain of TET enzyme to a promoter sequence to be demethylated.

Chen and colleagues generated chimeric constructs where the gene sequence of TET1, TET2, or TET3 catalytic domains was fused upstream with the gene sequence of specific ZF domains, which bind either specific CpG sites in ICAM-1 (CD54 zinc fingers) or EpCAM (ZFb zinc fingers) genes’ promoter sequences. Upon expression of the above constructs in human A2780 ovarian cancer cells, methylation levels of targeted CpG sites decreased, with the highest level of reduction reached by the TET2 catalytic domain. At the same time, this latter was able to increase mRNA levels of the *ICAM-1* gene but not those of *EpCAM* gene, even though targeted CpG sites’ demethylation was achieved in the EpCAM promoter. Expression of constructs with mutated TETs’ catalytic domains did not either exert demethylation of CpG sites or increase transcription of ICAM-1, thus corroborating the quality and the specificity of the system [35].

Together with TALE repeats and ZF domains, CRISPR-Cas9 technology has also been used to demethylate specific gene sequences.

Xu et al. used CRISPR-Cas9 system to induce directed demethylation on *RANKL*, *MAGEB2*, and *MMP-2* gene sequences, which are normally hypermethylated in HEK-293 cells. Specifically, the researchers introduced D10A and H84A substitutions in Cas9 to generate dCas9. This latter was then fused with the catalytic domain of Tet1 (namely Tet-CD) through a flexible linker. Such a construct was designated as dCas9-Tet1-CD. At the same time, in the same vector, two MS2-RNA elements (from MS2 phage) were inserted into the sgRNA transcription system. In addition, an MS2-Tet1-CD fusion protein with a flexible linker was generated by fusing the MS2 coat protein with Tet1-CD. By specific sgRNAs, both dCas9-Tet-CD and MS2-Tet1-CD could be recruited to a target locus. *RANKL* gene sequence underwent demethylation in HEK-293FT cells—a variant of HEK-293 [37]—and its transcript levels increased when the chimeric protein was expressed together with specific sgRNAs (sgR3 and R8). Indeed, *RANKL* gene transcript level was upregulated because the methylation status of this locus decreased. Similar results were obtained with a cell line of neuroblastoma, SH-SY5Y cells, meaning that the system was not cell-type specific. In addition, transcript levels of MAGEB2 in HeLa cells and MMP-2 in HEK-293FT cells were also increased by using the above constructs plus gene-specific sgRNAs, meaning also that the system used was not gene locus-specific [36].

Active DNA demethylation also correlates with DNA damage. Indeed, it was shown that Single Strand Breaks (SSBs) could accumulate at the level of neuronal enhancers of post-mitotic neurons, in the proximity of CpG sites and sites of DNA demethylation [38]. Moreover, these specific SSBs correlated with active DNA demethylation promoted by TET enzymes plus TDG, as recently shown by Wang and colleagues in Induced Pluripotent Stem Cells (iPSCs)-derived Neurons (iNs). To this aim, the researchers knocked out TDG in iPSCs, which were then induced to iNs or degraded TDG, via a degron, after inducing iPSCs to iNs. In both cases, Wang and colleagues found an accumulation of 5fC and 5CaC with fewer SSBs compared to the WT counterparts [39].

## 4. The Role of Non-Coding RNAs in DNMTs’ Activity: Impact on DNA Methylation

Recent studies have increasingly revealed the pivotal role of non-coding RNAs (ncRNAs), such as microRNAs (miRNAs) [40], in regulating the activity of DNA methyltransferases, which are key players in DNA methylation processes linked to various cancers and diseases. For example, Croce and his group found that levels of miRNA (miR)-29 family, including miR-29a, miR-29b, and miR-29c, were inversely correlated with the expression levels of DNMT3A and DNMT3B in non-small cell lung cancer (NSCLC) tissues. Specifically, transfection of each miR-29 member into A549 lung cancer cells led to a reduction in DNMT3A and DNMT3B mRNA levels, which correlated with decreased global DNA methylation and the re-expression, in both A549 and H1299 lung cancer cells, of tumor suppressor genes such as *FHIT* and *WWOX*, normally silenced through promoter methylation in lung cancer. Moreover, this miR-29 transfection induced apoptosis and reduced tumorigenesis in vivo in engrafted A549 tumors [41].

In AML cells, Garzon et al. demonstrated that miR-29b could induce global DNA hypomethylation by directly targeting DNMT3A and DNMT3B and indirectly regulating DNMT1 via the ZF transcription factor Sp1. This was evident in K562, MV4-11, and Kasumi-1 AML cell lines, where pre-miR-29b transfection or viral infection reduced DNMT3A, DNMT3B, and DNMT1 mRNA and protein levels, inducing global DNA demethylation, in MV4-11 and Kasumi-1 cells, similar to the effects of DAC. This global demethylation resulted in the re-expression of hypermethylated tumor suppressor genes, such as *ESR1* and *p15INK4b*, in AML [42].

Other studies have explored the role of miRNAs in regulating DNMTs through direct interactions. For example, Chen et al. (2011) investigated the effect of oxidized low-density lipoprotein (oxLDL) on primary human aortic smooth muscle cells (HASMCs), finding that oxLDL treatment increased the migration of these cells in a wound healing assay [43,44]. This was associated with an upregulation, both at transcript and protein levels as well as in activity, of Matrix Metalloproteinases (MMP-2 and MMP-9), whose gene expression was linked to decreased DNA methylation. Reduced DNA methylation also correlated with decreased DNMT3B mRNA and protein levels. In the end, the authors demonstrated that oxLDL treatment led to increased levels of miR-29b, which directly targeted DNMT3B, thus promoting DNA demethylation and enhancing HASMC migration. These findings suggest that targeting miR-29b or DNMT3B could be beneficial for treating cardiovascular diseases [45].

Similarly, in colorectal cancer (CRC), Chen et al. (2015) found that decreased levels of miR-124 and miR-506 were associated with aberrant DNA methylation (hypermethylation). Transfection of CRC cell lines SW480 and SW620 with miR-124 or miR-506 reduced either DNMT3B expression directly or DNMT1 expression indirectly (via Sp1 direct downregulation), leading to decreased proliferation, impaired wound closure, and enhanced sensitivity to DNA damage agents, such as cisplatin and 5-fluorouracil. Notably, these miRNAs also induced global DNA demethylation, which resulted in the re-expression of genes like *E-cadherin*, *p16*, and *MGMT* that are typically silenced in CRC due to hypermethylation [46].

Beyond miRNAs, direct interactions between other ncRNAs and DNMTs have been observed. Di Ruscio et al. (2013) identified a non-polyadenylated non-coding RNA, named extra-coding CEBPA (ecCEBPA), transcribed downstream of the polyadenylation site of the *CEBPA* gene, which directly binds DNMT1, preventing methylation of the *CEBPA* gene itself. This interaction was found to be crucial for maintaining *CEBPA* gene expression in leukemia cells. Knockdown of ecCEBPA in U937 leukemia cells resulted in increased methylation of the *CEBPA* gene distal promoter and decreased CEBPA mRNA levels, while ectopic expression of ecCEBPA in K562 cells led to decreased methylation of *CEBPA* gene distal promoter, codifying sequence, and 3′UTR as well as increased CEBPA transcript levels. Moreover, ecCEBPA was shown to bind directly to the catalytic domain of DNMT1, and this binding was specific to DNMT1 rather than to other DNMTs. The authors further demonstrated that ncRNAs—DNMT1 interaction occurred on thousands (4866) of gene loci, suggesting that such RNA–DNMT1 interactions could have a widespread role in modulating genomic DNA methylation [47].

Inspired by these findings, Esposito et al. (2022) developed aptamers derived from ecCEBPA, called aptaDirs, which specifically bind DNMT1 and inhibit its methylation activity. These aptaDirs were shown to effectively inhibit DNMT1 activity in vitro and in K562 cells, leading to hypomethylation of numerous CpG islands and increased CEBPA mRNA levels. Furthermore, in vivo experiments in immunodeficient NSG mice with K562 tumor xenografts showed that aptaDir treatment reduced tumor volume, promoted DNA demethylation, and increased transcription of different genes. In addition, DNMT1 methylation inhibition via aptaDir treatment was shown to be reversible, which made the authors conclude that this new strategy could be a very great asset in clinics [48].

Lastly, in the context of myeloid cells, Jones et al. (2021) identified the long intergenic non-coding (Linc) RNA CCDC26, which (directly or indirectly) binds DNMT1 and retains it in the nucleus. Knocking out CCDC26 in K562 cells led to DNMT1 mis-localization to the cytoplasm, resulting in reduced DNA methylation and the re-expression of genes normally silenced by DNMT1 activity. This study highlights how LincRNA CCDC26 contributes to maintaining DNMT1 function and nuclear localization, and its loss can lead to DNMT1 mis-localization with global DNA demethylation [49].

Together, all the above studies emphasize the diverse mechanisms through which ncRNAs modulate DNMTs activity and DNA methylation, providing valuable insights into potential therapeutic avenues for cancers and diseases driven by epigenetic dysregulation.

## 5. Pharmacological Inhibition of DNA Methylation in Biomedical Research: Pros and Cons

Pharmacological inhibition of DNA methylation via AZA and DAC has been widely used in the clinic throughout the years to specifically treat myelodysplastic syndromes (MDSs) and hematological cancers, upon FDA approval [50,51], such as AML [51,52]. Indeed, both AZA and DAC were, overall, able to slowdown neoplastic transformation of MDS and increase survival in patients [53,54,55,56]. However, due to the capability of AZA and DAC to be incorporated into nucleic acid structures (see paragraph 2), the outcomes in cells can be several. Indeed, Jackson-Grusby et al. found that DAC can induce mutations at CpG dinucleotides, which can thus question the benefit for oncological patients [57]. DAC toxicity was also primarily shown to be related to DNA methyltransferase trapping and, thus, its consequential inhibition, rather than DNA demethylation itself [58]. Interestingly, low doses of either DAC or AZA were shown to exert antitumor effects on leukemia cells, also impacting self-renewal [59]. These antitumoral effects can be explained by the reactivation of tumor suppressor genes (TGSs), which are silenced due to DNA hypermethylation in cancer [60,61]. Indeed, both DAC and AZA have been shown to re-induce the expression of tumor suppressor genes *FOXO1* and *GADD45γ*, respectively, in MDSs [62,63]. Both drugs have also been recently shown to demethylate *CDKN2A* and *CDKN2B,* with the following re-expression of p16 and p15 proteins in HCT116 cells [19].

Apart from the re-expression of TGSs, pharmacological inhibition of DNA methylation could also result in oncogene suppression. It was demonstrated that gene body methylation is associated with increased levels of gene transcription [64,65,66] and, indeed, Yang et al. found that DAC caused gene body demethylation in genes being part of cellular pathways regulated by c-MYC, in HCT116 cells [67]. Thus, it is clear why inhibition of DNA methylation via drugs is the most widely used strategy in biomedical research, and more and more effort must be made to guarantee its benefits for oncological patients.

A brief table summarizing some of the DNA methylation-inhibiting drugs used in biomedical research is displayed (Table 1).

However, despite their confirmed anticancer activity, it has also been shown that DNA demethylating drugs can favor metastasis dissemination. In this regard, recently Le and colleagues have demonstrated that increasing doses of AZA can induce metastasis in Hepatocellular Carcinoma (HCC) cells. Specifically, using trans-well assay, the researchers found that increasing doses of AZA let HCC cells migrate faster compared to lower doses. Moreover, administration in nude mice of AZA allowed HCC cells, injected intravenously, to form metastasis in the lungs. This was correlated to the increased expression of both transcript and protein levels of *RDH16*, a gene coding an enzyme involved in retinoic acid (RA) synthesis, which is normally hypermethylated and, thus, silenced in HCC cells. Indeed, overexpression of a lentiviral vector harboring *RDH16* cDNA was able to increase HCC cell migration as well as metastasis formation. The authors stated that *RDH16* gene expression can influence the expression of oncogenes or tumor suppressor genes via the activity of RA, which can thus explain the metastatic capacity of HCC cells upon AZA treatment [68].

DAC treatment has also been shown to enhance tumoral cell invasion via the increased expression of Matrix Metalloproteinase-1 (MMP-1). Indeed, Poplineau and colleagues, by treating HT1080 fibrosarcoma cells with DAC, found that MMP-1 mRNA and protein levels were increased compared to the untreated counterpart, and this correlated with a higher invasion potential. Particularly, *MMP-1* gene’s promoter methylation status was not prominently affected by DAC; rather, *MMP-1* increased gene expression was related to increased recruitment of Sp1 and Sp3 transcription factors at *MMP-1* gene’s promoter in a context of permissive chromatin (increased H4ac and decreased H3K273me levels) [69].

Even though AZA is well employed as treatment in MDS, its administration in patients could enhance the invasiveness of cancer cells. Indeed, Bernal and colleagues found that treatment with AZA of MOLM-13 cells, a model of acute myeloid leukemia derived from MDS, increased cells’ invasiveness in a matrigel assay. This was related to decreased DNA methylation levels of the *Matrix Metalloprotease*-9 (*MMP-9*) gene and a concomitant increase of both its mRNA levels and protein activity, with the latter also displayed in MDS patients after 6 cycles of AZA treatment [70].

From what is shown above, it is thus clear that extreme caution is envisaged in treating oncological patients with DNA demethylating drugs.

## 6. Conclusions

In this manuscript, we have given a brief glimpse into the most common strategies (pharmacological/genetic inhibition/perturbation) as well as the newest ones (CRISPR-Cas9; AID, aptaDirs) employed to achieve global as well as targeted DNA demethylation. We have also glimpsed into the regulation of DNMTs’ activity by non-coding RNAs as well as the use of DNA-demethylating drugs in biomedical research.

Studying the effects of DNA demethylation has a pivotal importance in understanding cellular processes such as development, as well as tumorigenesis, and thus it is clear that specific strategies to achieve and control DNA demethylation are needed.

While the use of demethylating agents can have a pleiotropic effect on cell viability, targeted active [34,35,36] as well as passive and inducible [27,28,29] DNA demethylation strategies have been revealed as powerful tools to study the loss of 5mC, globally or on specific gene loci, with the absence of important side effects. Lastly, combining targeted as well as inducible DNA demethylation with no side effects can be a great asset in paving the way toward precision medicine.

## Figures and Tables

**Table 1:** Most common DNA-demethylating drugs used in biomedical research.

DNA Demethylating Drugs	Malignancies	Type
5-Azacitidine (AZA)	AML, MDS, Solid tumors	Nucleoside analog
5-aza-2′-deoxycytidine (DAC or decitabine)	AML, MDS	Nucleoside analog
GSK3685032	AML	Non-nucleoside
Guadecitabine (SGI-110)	AML, MDS	Nucleoside analog
5-azacytidine-5′-elaidate (CP-4200)	AML	Nucleoside analog
MG98	Solid tumors	Oligonucleotide Antisense
NSC309132 (or Zebularine)	Solid tumors	Nucleoside analog
Procainamide	Solid tumors	Non-nucleoside
Epigallocatechin-3-gallate (EGCG)	Solid tumors	Non-nucleoside
SGI-1027	Leukemia	Non-nucleoside
Hydralazine	Breast, ovarian, cervical cancers	Non-nucleoside

## Data Availability

Not applicable.
